# The variation profile of intestinal microbiota in blunt snout bream (*Megalobrama amblycephala*) during feeding habit transition

**DOI:** 10.1186/s12866-018-1246-0

**Published:** 2018-09-03

**Authors:** Jin Wei, Xianwu Guo, Han Liu, Yuanyuan Chen, Weimin Wang

**Affiliations:** 1 College of Fisheries, Key Lab of Agricultural Animal Genetics, Breeding and Reproduction of Ministry of Education/Key Lab of Freshwater Animal Breeding, Ministry of Agriculture, Huazhong Agricultural University, Wuhan, 430070 Hubei China; 2 Lab of Biotecnología Genómica, Centro de Biotecnología Genómica, Instituto de Politécnico Nacional, Boulevard del Maestro S/N esq. Elías Piña, Col. Narciso Mendoza, C.P. 88710, Cd, Reynosa, Tamaulipas Mexico; 3 College of Fisheries, Huazhong Agricultural University, Wuhan, 430070 People’s Republic of China

**Keywords:** Blunt snout bream, Gut microbiota, Feeding habit transition, Core microbiome, Enzyme activity, Cellulose-degrading bacteria

## Abstract

**Background:**

The blunt snout bream (*Megalobrama amblycephala*) is one of the most important commercial herbivorous fish in China, and dietary transition is an important event in blunt snout bream development. Gut microbiota has a vital role to host animal. However, little was known about the relationship among feeding habits transition, gut microbiota and digestive enzymes of gut content.

**Results:**

In this study, 186,328 high-quality reads from nine 16S rRNA libraries were obtained using the Illumina MiSeq PE300 platform. The valid sequences were classified into 388 Operational Taxonomic Units, and a total of 223 genera, belonging to 20 phyla, were identified. The clustering result of gut bacterial communities is consistently related to the clustering result of intestinal content compositions. *Proteobacteria* and *Firmicutes* constitute the ‘core’ gut microbiota of blunt snout bream. *Cetobacterium* and *Rhizobium* were identified as microbiological markers of gut microbiota at zooplankton-based diet stages and diet transition stages, respectively. Moreover, thirteen potential cellulose-degrading bacteria were detected in our study. The canonical redundancy analysis (RDA) revealed that the feeding habits strongly influenced the gut microbiota and the digestive enzyme activities of gut content, while the result of PICRUSt test suggests that the metabolic capacity of gut microbiota was affected by feeding habit.

**Conclusions:**

This study provided a comprehensive survey of the gut microbiota in blunt snout bream during its dietary transition period for the first time and clearly showed that the gut microbiota was strongly affected by feeding habit. This work allows us to better understand the relationship among gut microbiota, nutrition metabolism and feeding habits in vertebrate. Further, our study provides a reference for future studies investigating the metabolic adaption of herbivorous fish to shift to a vegetarian diet during their life history.

**Electronic supplementary material:**

The online version of this article (10.1186/s12866-018-1246-0) contains supplementary material, which is available to authorized users.

## Background

The vertebrate gastrointestinal tract, which harbors diverse and abundant bacteria, archaea, fungi and viruses, is a complex microbial ecosystem [1, 2]. The gut microbiota can be regarded as an additional ‘organ’ of the host animal with beneficial effects on metabolic capacity, energy utilization and storage, immune function and health maintenance [1–3]. In fish, the structure, composition and ecological function of gut microbiota are greatly influenced by the factors from host (e.g. genetics, weight, gender, development, immunity, trophic categories and metabolic capacity) [4–8], environment (e.g. water, diet and medicine) [9–11], and showed circadian fluctuation and individual variations [1, 12]. In addition, bacterial colonization of fish intestines begins at the larval stage [1, 12], during which none of these factors may be as important in the development of the microbiota as feeding habit [13, 14]. For instance, significant influences of first feeding on development of the gut microbiota were reported in turbot and rainbow trout start-feeding larval [15, 16].

Fish have specific intestinal microbiota consisting of aerobic, facultative anaerobic and obligate anaerobic bacteria [1, 2, 17]. *Proteobacteria*, *Firmicutes* and *Actinobacteria* identified in some freshwater fish are considered to constitute the ‘core’ intestinal microbiota [1, 18–22]. Cellulose is one of the most important forage resources for herbivorous fish. Some strains belonging to *Clostridium*, *Methylobacterium*, *Enterobacter*, *Citrobacter*, *Bacillus* and *Pseudomonas* isolated from the gastrointestinal tract of fish were identified as the potential cellulose-degrading bacteria [22–25]. Chitin, as the world’s second abundant biomass following cellulose, is a major body component of zooplankton [26]. Thus, digestion of chitin is vital for the fish, which feed on zooplankton [25, 27]. Some strains belonging to *Aeromonas*, *Vibrio*, *Plesiomonas*, *Acinetobacter*, *Flavobacterium* and *Bacillu*, which were isolated from the digestive tract of Atlantic salmon, Dover sole, Japanese flounder, tilapia, respectively, were identified as the chitinase-producing bacteria [25, 28–30].

Blunt snout bream (*Megalobrama amblycephala*), popularly named Wuchang fish, is herbivorous, freshwater fish endemic to China [31]. Bream show several favorable characteristics including a diet of natural foods, fast growth, tender flesh and high disease resistance [32, 33]. The aquaculture of blunt snout bream has expanded rapidly in recent years. The total production of bream reached approximately 826,178 tons and ranked sixth in total freshwater fish production in China in 2016 [34]. Blunt snout bream have been reported to experience dietary transition. The fry, under 3.1–3.5 cm in body length, lives on zooplankton, such as *Protozoa*, *Rotifera*, *Cladocera* and *Copepoda*. The transition from a carnivorous to an herbivorous diet begins when the fish reaches 3.4–3.7 cm in body length, and then the fish maintains the feeding habit for the rest of life, feeding on aquatic plants such as *Lemna minor*, *Vallisneria natans* and *Hydrilla verticillata* [35, 36].

In the past decades, traditional microbiological techniques (isolation and cultivation) and 16S rRNA gene fingerprinting methods (denaturing gradient gel electrophoresis (DGGE) and terminal restriction fragment length polymorphism (T-RFLP)) were performed to characterize the microbial community. In recent years, next-generation sequencing (NGS) of 16S rRNA gene, which greatly facilitated the ability to profile microbial communities at high resolution in complex environments, was increasingly applied in the research on gut microbiota composition of several fish species. In the present study, next-generation sequencing (NGS) of 16S rRNA gene was used to research the dynamics of intestinal microbiota during the dietary transition of blunt snout bream. This work was designed to investigate the dietary stage-specific intestinal microbiota composition and assess the potential roles of gut bacteria in the digestion of blunt snout bream.

## Methods

### Diets, rearing conditions and sampling

To reduce the potential impact of host genotype variation, all experimental blunt snout bream (*n* = 20,000) consisted solely of offspring from a single pair of mating fish, which were collected from Liangzi Lake (Hubei, China). These artificially bred cohort members were divided evenly into three groups in independent tanks at 26 ± 2 °C and were raised under conditions that simulated their natural aquatic environment. The fish were exposed to a daily cycle of 12 h of light and 12 h of dark. Sufficient zooplankton and aquatic plants were put into each tank during the first 56 days, so that the fish were fed to satiation and had free access to different edible food at different stages of feeding habits (voluntary feeding behavior). The sediments from each tank were removed on the 57th day post fertilization (d.p.f.), and the fish were fasted for 3 days. Subsequently, these fish were only fed with aquatic plants. The zooplanktons, which consisted of *Rotifera* (a mixed culture of *Brachionus angularis*, *Brachionus calyciflorus* and *Filinia longiseta*), *Cladocera* (a mixed culture of *Moina micrura* and *Diaphanosoma brachyurum*), *Copepoda* (*Sinocalanus tenellus*) and brine shrimp (*Artemia franciscana*), were supplied by Aquaculture Base of College of Fisheries, Huazhong Agricultural University in Wuhan City, Hubei Province, China. The aquatic plants, including *L. minor* and *H. verticillata*, were exclusively collected from the South Lake, Wuhan, China.

The continuous observation was conducted throughout multiple key stages of dietary transitions. We examined the dissected intestines of 10 individuals sampled randomly from each of the three replicate tanks, under optical microscopy every day to monitor the dietary component change. The samples were collected when a significant change in the composition of intestinal contents was noticed. The weight and standard length of each individual were measured as metrics of individual fish development and growth before dissection [7]. The intestinal contents were identified based on the morphological characteristics of prey items, and quantitative features (numbers and proportions) of these items were examined by the point method [37, 38]. The proportions of each prey item in intestinal contents were compared by the Kruskal-Wallis H test followed by the Nemenyi test.

**Fig. 1:**
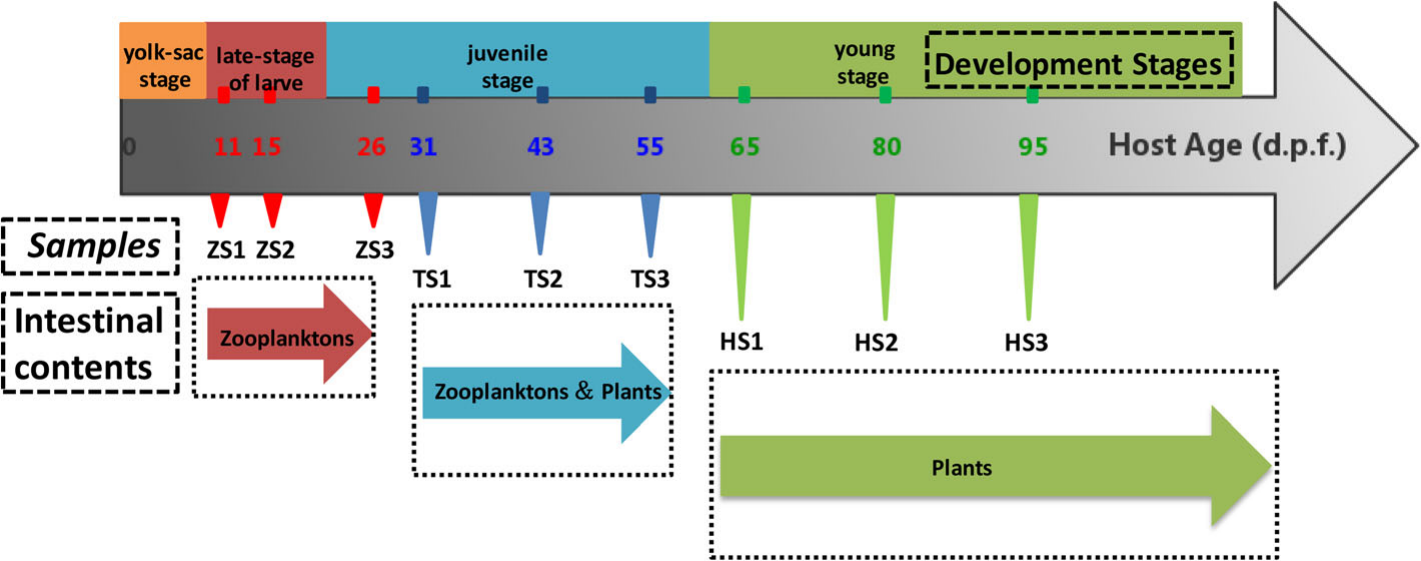
Flow chart of blunt snout bream development and experimental design. The flow chart shows the important developmental stages of individual (top), the major constituent of intestinal contents (bottom), the time nodes of sampling (gray arrow) and the sample names

Development of blunt snout bream feeding habits can be broadly divided into three stages: the zooplankton-based diet stage (ZS), the diet transition stage (TS) and the herbivorous diet stage (HS) in this study. The diet transition stage was marked by the first appearance of ingested aquatic plants in the intestines. The three stages of feeding habits can be further divided into nine sub-stages based on the food composition as a whole: ZS1, ZS2, ZS3, TS1, TS2, TS3, HS1, HS2 and HS3. Figure 1 provides detailed information on sampling. Ward’s clustering method using Euclidean distances was then performed to classify sampled individual variations in diet into several cluster groups by SPSS v22.0 (IBM, Inc., Armonk, NY) [38].

The sampling was performed in a manner that minimizes cross-contamination of samples, non-intestinal microorganism, tanks and time points. The blunt snout bream was collected from the tanks approximately at the same time each day (between 9:00 to 9:30 am) and then transferred to a clean bench. After anesthetized with 100 mg L − 1 MS-222 (Sigma-Aldrich, St Louis, MO, USA), the following operation was performed on the ice. The entire intestine from the end of esophagus to the cloaca was dissected out. The dissection was performed on a separate, sterile glass slide (larvae) or Petri dish cover (juveniles and young adults) under an anatomical lens by individual-use insect pins [7, 39]. The gut content from the midgut region to the hindgut region was collected and mixed thoroughly in a sterile fashion. Due to very few intestinal contents (less than 50 mg) obtained from individual fish, each sample represents a blend of contents from more than 30 individual fish collected at the same time point. Individual differences were eliminated efficiently by using pooled samples, and these samples were sufficient for both DNA extraction and enzyme activity determination. The fresh gut contents were aseptically squeezed into sterilized microtubes and homogenized, then stored at − 80 °C within 15 min for further microbiome and enzyme activity analyses.

Experimental procedures were conducted in accordance with the guidelines for the care and use of animals for scientific purposes set by the Ministry of Science and Technology, Beijing, China (No. 398, 2006) and the Institutional Animal Care and Use Ethics Committee of The Scientific Ethic Committee of Huazhong Agricultural University (Permit Number: HZAUFI-2016-006).

### DNA extraction, amplicon library preparation and sequencing

DNA extraction was performed within two days after dissection to minimize the potential effect of external environment variation on intestinal microflora and to prevent DNA degradation. The bacterial genomic DNA was extracted from 200 mg of contents using the QIAamp DNA Stool Mini Kit (Qiagen, Germantown, MD) following the manufacturer’s protocol. The concentration and purity of total DNA were determined using the NanoDrop 2000 Spectrophotometer (Thermo Fisher Scientific, Wilmington, DE). PCR reactions were used to amplify the V3-V4 region of bacterial 16S rRNA genes using primers 338F-806R: 338F: ACTCCTACGGGAGGCAGCA; 806R: GGACTACHVGGGTWTCTAAT [40]. PCR products were extracted from 2% agarose gels and purified using the AxyPrep DNA Gel Extraction Kit (Axygen Biosciences, Union City, CA, U.S.) following the manufacturer’s manual. The purified PCR products were quantified via QuantiFluor™-ST (Promega, U.S.). The DNA pool was generated from the purified products in equimolar ratios to a final concentration. The pooled products were submitted to Shanghai Majorbio Bio-pharm Technology Co., Ltd. (Shanghai, China) for paired-end sequencing (2 × 300) with an Illumina MiSeq PE300 platform following the standard protocols. The sequence data that were generated in the current study have been deposited in the NCBI Short Read Archive (SRA) with the accession numbers SRR3452703, SRR3452705, SRR3452706, SRR3452708, SRR3452709, SRR3452710, SRR3452711, SRR3452714 and SRR3452715.

### 16S rRNA gene sequence processing and statistical analysis

The raw fastq files of Illumina reads were demultiplexed and quality filtered using QIIME 1.6.0 [41] with previously described criteria [42]. UPARSE (version 7.1 http://drive5.com/uparse) [43] was applied to cluster the Operational Taxonomic Units (OTUs) at an identity threshold of 97%, and UCHIME was utilized to identify and remove chimeric sequences [44]. The phylogenetic affiliation of each 16S rRNA gene sequence was assessed by RDP Classifier (http://rdp.cme.msu.edu) against the SILVA (SSU115) 16S rRNA database with a confidence threshold of 70% using the default settings.

Sequencing effort was equalized by randomly subsampling the reads to an equal sequencing depth for each sample (equal to the sample with the smallest number of reads) [45]. OTUs, which reached a 97% nucleotide similarity level, were used for alpha diversity (Shannon index, Simpson index and Evenness), phylogenetic beta diversity measures (hierarchical cluster tree), richness (Ace and Chao1 estimator), Good’s coverage, Venn diagram, Shannon-Wiener curve and rarefaction curve analysis using Mothur 1.30.1 [46]. The rank-abundance curves and species accumulation curves were produced using vegan [47] packages in the R (version 3.1.2) statistical environment (R Core Team, 2014).

To study the global condition of intestinal microbiota composition in blunt snout bream at three stages of feeding habits, the sequencing data obtained from microbial communities at nine sub-stages of feeding habits were merged into three aggregate datasets (ZS1, ZS2 and ZS3 were merged into group ZS; TS1, TS2 and TS3 were merged into group TS; HS1, HS2 and HS3 were merged into group HS). Several analyses, including rank-abundance curve, bacterial composition analyses, were also performed on the aggregate datasets of three stages of feeding habits. To identify the specific bacterial taxa (microbiological markers) associated with feeding habits, the gut microbiota of fish in three diet-stage groups were compared using linear discriminant analysis effect size (LEfSe) method (http://huttenhower.sph.harvard.edu/lefse) [48]. A significance level (alpha value) of 0.05 and a significance threshold of 3 were used for all biomarkers analyzed in this study. PICRUSt algorithm [49] was performed to predict the metabolic activity of bacterial communities found in the gut contents of fish at different diet stages, and functional inferences were made from the Kyoto Encyclopedia of Gene and Genomes (KEGG) catalog. The pathway functions were categorized at levels 2 and 3.

All tests for significance were two-sided, and *p* values< 0.05 were considered statistically significant. In addition, the intestinal microbiota of three diet-stage groups were compared by the Kruskal-wallis H test followed by Nemenyi test at different taxon levels.

### Enzyme activity determination and analysis

Amylase activity was measured according to starch hydrolysis using the Somogyi-Nelson colorimetric method (modified by Robyt and Whelan [50]), and it was expressed as mg of maltose released within one min per gram of sample (humid intestine/contents), mg/g/min. Cellulase activity was determined using the methodology described by Saha and Ray [51] (modified from Sadasivam and Manickam [52]), and it was expressed as mg of glucose liberated per gram of sample (tissue/contents) per hour at 37 °C. Lipase activity was estimated in accordance with the method of Bernfeld [53], and one unit of lipase activity was expressed as the amount of enzyme that released one μmol of free fatty acid per minute at 37 °C. Chitinase activity was determined according to the procedure described by Danulat and Kausch [54], and it was defined as mg of N-acetyl-D-glucosamine liberated within one hour per gram of sample (tissue/contents) at 37 °C. Enzyme activity was assayed in technical triplicate. Synergy™ 2 multi-detection microplate reader (BioTek, America) was substituted for an ordinary spectrophotometer to determine the absorbance of enzyme extract because only a thimbleful of intestinal contents could be utilized for the enzyme assays.

To better understand the relationship between gut microbial composition and the enzymes activities of gut content in fishes at different stages of feeding habit, the canonical redundancy analysis (RDA) was conducted with the vegan package in R. Specifically, the significance was assessed by permutation tests, and the Pearson correlation coefficient was estimated with the bioenv function (vegan package).

## Results

### Classification of intestinal contents

The intestinal contents were examined throughout the course of blunt snout bream development, and the prey organisms were classified into six categories: *Rotifera*, *A. franciscana*, *Copepoda*, *Cladocera*, *L. minor* and *H. verticillata*. Significant differences in intestinal content composition were found among the nine sub-stages (Additional file 1) (*p* < 0.05, Kruskal-Wallis H test). The variations of gut content composition among individual fish were classified into three cluster groups by Ward’s linkage cluster analysis. Interestingly, the intestinal content compositions at stage ZS3 were grouped with those at stage TS1, but separated from those at stages ZS1 and ZS2 (Additional file 2).

### Characteristics of sequencing data and taxonomy

A total of 294,674 valid reads with a median read length of 434.62 bp (ranging from 431.88 to 445.49 bp) were generated from nine samples. Following quality trimming and chimera detection, 186,328 high-quality reads remained, accounting for 63.2% of the valid reads with an average of 20,703 reads (ranging from 15,995 to 29,924) per barcoded sample for further analysis. Sequencing effort was equalized by randomly subsampling all samples to an equal sequencing depth (15,995 reads per sample). The Good’s coverage for the observed OTUs was 99.79 ± 0.02% (mean ± S.E.) and the rarefaction curves (Additional file 3) showed clear asymptotes, which together indicate a near-complete sampling for the investigation of intestinal microbiota. The trend of the species accumulation curve revealed that the sampling quantity was sufficient for analysis (Additional file 4). Moreover, the microbial complexity of gut at different feeding habit stages was estimated on the basis of richness and alpha-diversity, and it showed distinct differences (Table 1).

**Table 1:** Overview of quality sequences, richness and alpha-diversity index of gut microbiota at different feeding habit stages

Diet stages	Total quality sequences	Number of OTUs	Richness estimator		Diversity index		Good’s coverage
			Ace	Chao 1	Shannon	Simpson	
ZS	78,837	133±27	175±31	174±28	2.18±0.29	0.21±0.04	99.79±0.02%
TS	55,052	241±8	281±11	283±7	3.13±0.22	0.14±0.03	
HS	52,439	211±13	235±17	236±17	3.13±0.14	0.09±0.01	

### The dominant bacterial taxa of intestinal microbiota **in three diet-stage groups**

**Fig. 2:**
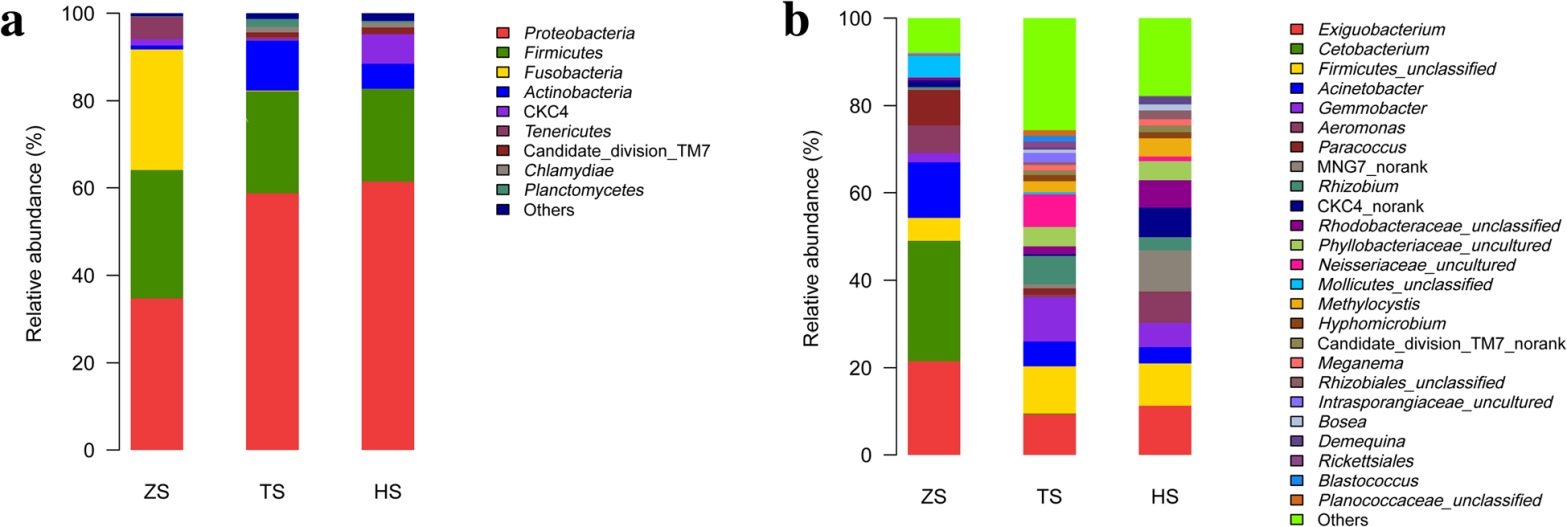
Composition of gut microbiota in blunt snout bream of three diet-stage groups. **a** Distribution of the major phyla among three diet groups. **b** Distribution of the major genera among three diet groups

A total of 223 genera, belonging to 20 phyla including four candidate divisions (TM6, TM7, CKC4 and OD1), were identified in this study. The most abundant taxa of bacteria were shown in Fig. 2. At the phylum level, the ZS group was numerically dominated by phyla *Proteobacteria* (34.71%), *Firmicutes* (29.38%), *Fusobacteria* (27.60%) and *Tenericutes* (4.90%), and these phyla represented 96.59% of the total bacterial sequences. The TS group was dominated by phyla *Proteobacteria* (58.74%), *Firmicutes* (23.42%) and *Actinobacteria* (11.34%), representing 93.51% of the reads. The HS group was dominated by *Proteobacteria* (61.43%), *Firmicutes* (21.24%), CKC4 (no-ranked) (6.77%) and *Actinobacteria* (5.67%), accounting for 95.11% of the reads (Fig. 2a).

At the genus level, the ZS group was numerically dominated by genera *Cetobacterium* (27.57%), *Exiguobacterium* (21.51%), *Acinetobacter* (12.68%), *Paracoccus* (8.16%), *Aeromonas* (6.34%), *Gemmobacter* (2.12%) and some unclassified genera, accounting for 90.03% of the reads. The TS group was dominated by genera *Gemmobacter* (10.02%), *Exiguobacterium* (9.33%), *Rhizobium* (6.50%), *Acinetobacter* (5.69%), *Methylocystis* (2.52%), *Paracoccus* (1.41%), *Hyphomicrobium* (1.41%), *Blastococcus* (1.33%) and some unclassified genera, representing 69.86% of the reads. The HS group was dominated by *Exiguobacterium* (11.26%), *Aeromonas* (7.03%), *Gemmobacter* (5.47%), *Methylocystis* (4.20%), *Acinetobacter* (3.71%), *Rhizobium* (3.05%), *Hyphomicrobium* (1.42%) and some unclassified genera, which represented 81.81% of the reads (Fig. 2b).

### Variations of gut microbiota in different feeding-habit stages

The diversity and richness of bacterial communities remained low at ZS1 and ZS2 stages, then increased rapidly from stage ZS3, peaked at stage TS3 and gradually decreased during the herbivorous stages (Table 1). As shown in Additional file 5, the most centralized distribution of OTUs was observed in stage ZS1, while the most diversified distribution of OTUs was observed in stages TS1 and TS2. The ZS group possessed a significantly lower intestinal microbiota diversity (the number of OTUs and Shannon’s diversity index) compared with the other two diet-stage groups (*p* < 0.05). Similarly, the ZS group possessed significantly lower richness indexes (Ace and Chao1) compared with the other two groups. Additional file 6 showed that the community evenness of the ZS group was relatively lower than the other two diet-stage groups.

**Fig. 3:**
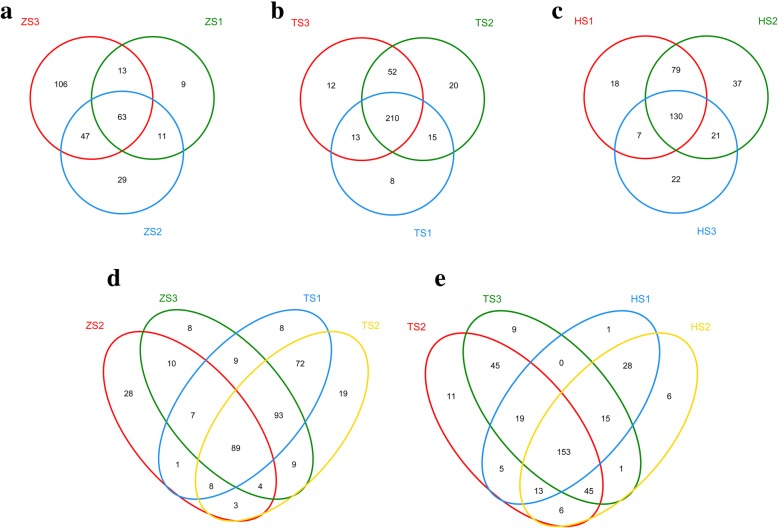
Venn diagrams illustrating the overlap of OTUs in intestinal microbiota. **a**, **b** and **c** The overlap of OTUs in intestinal microbiota among three sub-stages of each diet groups (ZS, TS and HS). **d**, **e** The overlap of OTUs in intestinal microbiota among the six sub-stages (ZS2, ZS3, TS1, TS2, HS1 and HS2) of diet transition process

The Venn diagram was used to illustrate the shared or unique OTUs. A total of 278, 330, and 314 OTUs were observed in ZS, TS and HS diet-stage groups, respectively. The ZS1, ZS2 and ZS3 libraries shared 63 of the 278 (22.7%) ZS OTUs (Fig. 3a), the TS1, TS2 and TS3 libraries shared 210 of the 330 (63.6%) TS OTUs (Fig. 3b), and Fig. 3c showed that the HS1, HS2 and HS3 libraries shared 130 of the 314 (41.4%) HS OTUs. This result suggests that the OTU composition changed dramatically in the ZS group and remained relatively stable in the TS group. Figure 3d showed that 110 of 269 (40.9%) OTUs were shared between ZS2 and ZS3 libraries, 198 of 318 (62.3%) OTUs were shared between ZS3 and TS1 libraries, 262 of 322 (81.4%) OTUs were shared between TS1 and TS2 libraries. Additionally, 187 of 334 OTUs (56.0%) were shared between TS3 and HS1 libraries, 209 of 292 OTUs (71.6%) were shared between HS1 and HS2 libraries, and 151 of 296 OTUs (51.0%) were shared between HS2 and HS3 libraries (Fig. 3e). This result illustrates that the most dramatic change of OTU composition occurred between ZS2 and ZS3 communities, not between ZS3 and TS1 communities or between TS3 and HS1 communities.

**Fig. 4:**
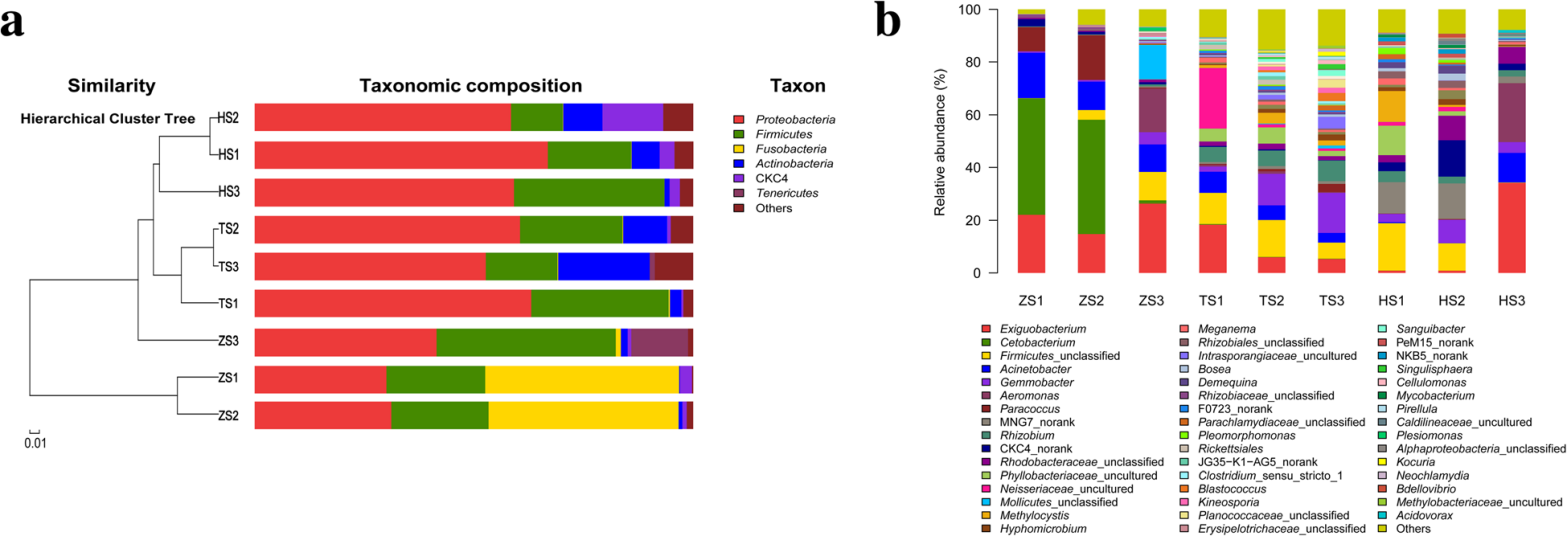
Composition of gut microbiota in blunt snout bream during different feeding habit sub-stages. **a** Distribution of the major phyla among nine feeding habit sub-stages and the hierarchical cluster tree of samples based on the composition of intestinal microbiota. Bray-Curtis community similarity index is used to deal with the community similarity and cluster analysis. **b** Distribution of the major genera among nine feeding habit sub-stages

When representative bacteria were classified into phyla, the variations of microbial community composition were observed among different feeding habit sub-stages. As shown in Fig. 4a, *Proteobacteria* and *Firmicutes* were widely present in the gut microbiota at nine diet sub-stages. The phylum *Actinobacteria* was present at low abundance in ZS1 and ZS2 communities (< 1%), but a marked increase was observed from stages ZS3 to HS2 (ranging between 1.5 and 16.4%), followed by a sharp decrease at HS3 (1.1%). Interestingly, the phylum *Fusobacteria* showed the highest abundance in ZS1 and ZS2 communities (44.2% and 43.4%) compared with the other seven communities (< 1%).

Figure 4b illustrated a detailed profile of the gut microbiota of nine feeding habit sub-stages at the genus level. *Exiguobacterium* and *Acinetobacter* were abundant in most sub-stages except HS1 and HS2, while *Gemmobacter* and *Rhizobium* were abundant in most sub-stages except ZS1 and ZS2. *Aeromonas* was relatively abundant at stages ZS3 and HS3, and *Methylocystis* was relatively abundant at stages TS1, TS2, TS3 and HS1. Interestingly, *Cetobacterium* and *Paracoccus* were particularly abundant in ZS1 and ZS2 communities yet were not consistently as abundant in the other seven communities.

### Difference and similarity of gut microbiota among three diet-stage groups

The changes in the gut microbiota of three diet-stage groups were assessed using the Kruskal-wallis H test followed by Nemenyi test at different taxon levels. These results are shown in Additional file 7. At the phylum level, *Proteobacteria* and *Firmicutes* were both extensive in all the diet-stage groups, while *Proteobacteria* was significantly more abundant in TS and HS groups (*p* < 0.05). ZS group possessed a significantly higher abundance of *Fusobacteria* but a significantly lower abundance of *Actinobacteria* compared with the other two diet-stage groups (*p* < 0.05). At the order level, *Rhodobacterales* were prevalent in all three diet-stage groups, while *Rhizobiales* were significantly less abundant in ZS group compared to TS and HS groups (*p* < 0.05). *Neisseriales* (class Beta-*proteobacteria*) and *Micrococcales* (phylum *Actinobacteria*) were significantly more abundant in TS group compared to the other two groups, while *Aeromonadales* (class Gamma-*proteobacteria*) was significantly less abundant in TS group (*p* < 0.05). *Pseudomonadales* (class Gamma-*proteobacteria*) and *Fusobacteriales* (phylum *Fusobacteria*) were enriched in the gut microbiota of ZS group but significantly decreased in abundance in TS and HS groups (*p* < 0.05). At the family level, *Rhizobiaceae* and *Neisseriaceae* were significantly more abundant in TS group compared with ZS and HS groups (*p* < 0.05). *Moraxellaceae* and *Fusobacteriaceae* were prevalent in the gut microbiota of the ZS group but significantly decreased in abundance in TS and HS groups (*p* < 0.05). MNG7 (candidate family of class *Rhizobiales*) were significantly more abundant in HS group compared with ZS and TS groups (*p* < 0.05). At the genus level, *Exiguobacterium* was the most abundant genus of gut microbiota in all diet-stage groups. *Cetobacterium* (order *Fusobacteriaceae*) was enriched in the gut microbiota of ZS group but significantly decreased in abundance in TS and HS groups (*p* < 0.05). TS group harbored a significantly greater proportion of *Gemmobacter* and *Rhizobium* in comparison with the other two groups (*p* < 0.05).

**Fig. 5:**
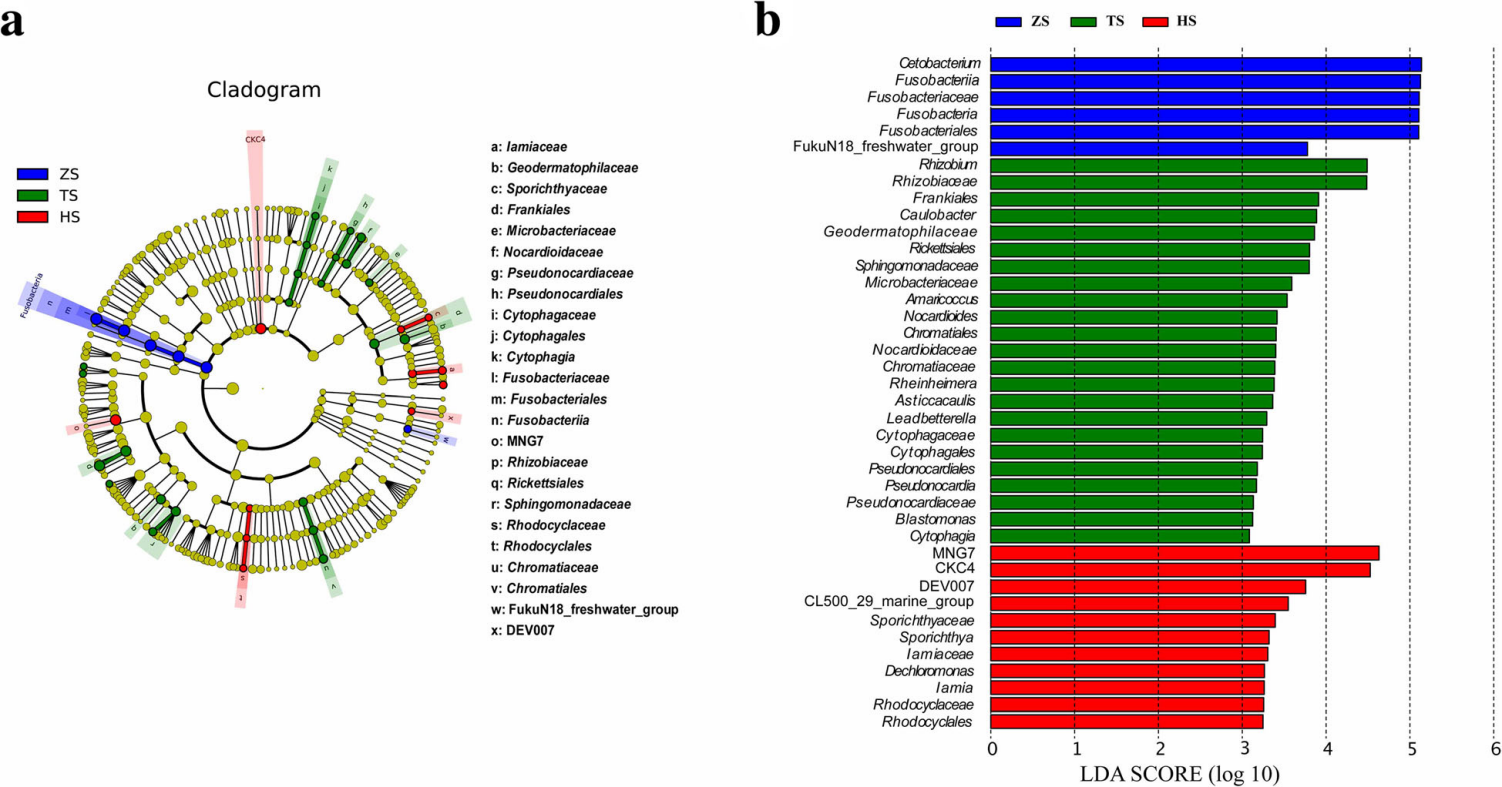
LEfSe identifying the most differentially abundant taxons among three feeding habit stages. **a** LDA effect size taxonomic cladogram comparing three aggregate datasets of 16S rRNA genes categorized by feeding habits. Significantly discriminant taxon nodes are colored and branch areas are shaded according to the effect size of taxa. (Blue) taxa enriched in ZS (zooplankton-based diet stage) group; (Green) taxa enriched in TS (diet transition stage) group; (Red) taxa enriched in HS (herbivorous diet stage) group. The corresponding nodes of taxa, which are not significantly differentially represented between sample groups, are colored yellow. **b** Taxa enriched in ZS (blue), TS (green) and HS (red) are indicated with LDA scores. Only taxa meeting an LDA significant threshold of 3 are shown

LEfSe emphasizes both statistical significance and biological relevance. Figure 5 showed the greatest differences in taxa between the three diet-stage groups, and several key phylotypes as microbiological markers were identified at different phylogenetic levels. For instance, *Cetobacterium* (belonging to family *Fusobacteriaceae*) could be used as a microbiological marker to differentiate ZS group from the other two groups, *Rhizobium* (belonging to *Rhizobiaceae*) could be used to differentiate TS group from the others, and MNG7 (candidate family of class *Rhizobiales*) could be used to differentiate HS group from the others.

**Fig. 6:**
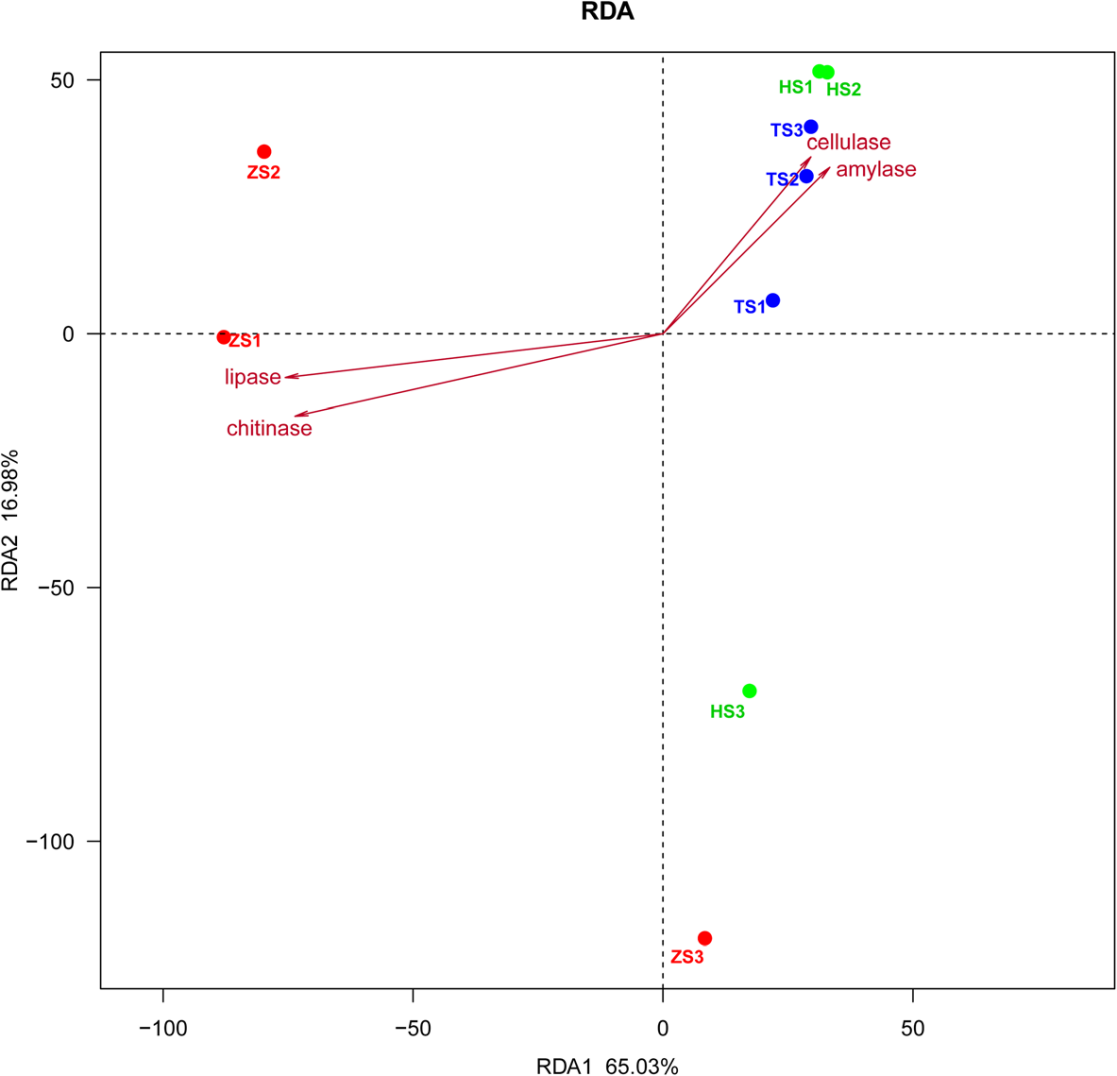
RDA plot showing the correlation among samples, microbial species and enzyme activities of gut content. The colored dots represent the samples of each feeding habit sub-stage. The inverted triangles represent the detected bacteria. The vectors represent the digestive enzyme activities, and the length and direction of the vector indicated the degree of correlation (the relative positive or negative relationship) between the digestive enzyme activities and the samples

The similarities of gut microbiota among different diet-stages were visualized by the hierarchical cluster analysis (Fig. 4a) and the canonical redundancy analysis (Fig. 6). Figure 4a showed a net separation of group TS (TS1, TS2 and TS3 communities), group HS (HS1, HS2 and HS3 communities) and part of group ZS (ZS1and ZS2 communities). Unexpectedly, the ZS3 community was separated from the group of ZS1 and ZS2 communities, but it clustered as one branch with groups TS and HS. This result suggests that ZS3 community was more similar to TS1 community. Similarly, Fig. 6 demonstrates that TS1, TS2 and TS3 communities grouped to the right of the graph along RDA 1, which accounts for 65.03% of the total variations. HS1 and HS2 communities were closely related to the group of TS, while ZS1 and ZS2 communities were separate, far from group TS along RDA1. By contrast, both ZS3 and HS3 communities were separate, far from all the groups along RDA 2, which accounted for 16.98% of the total variations.

### Predicted fish gut microbiota function using PICRUSt and cellulose-degrading bacteria analysis

**Fig. 7:**
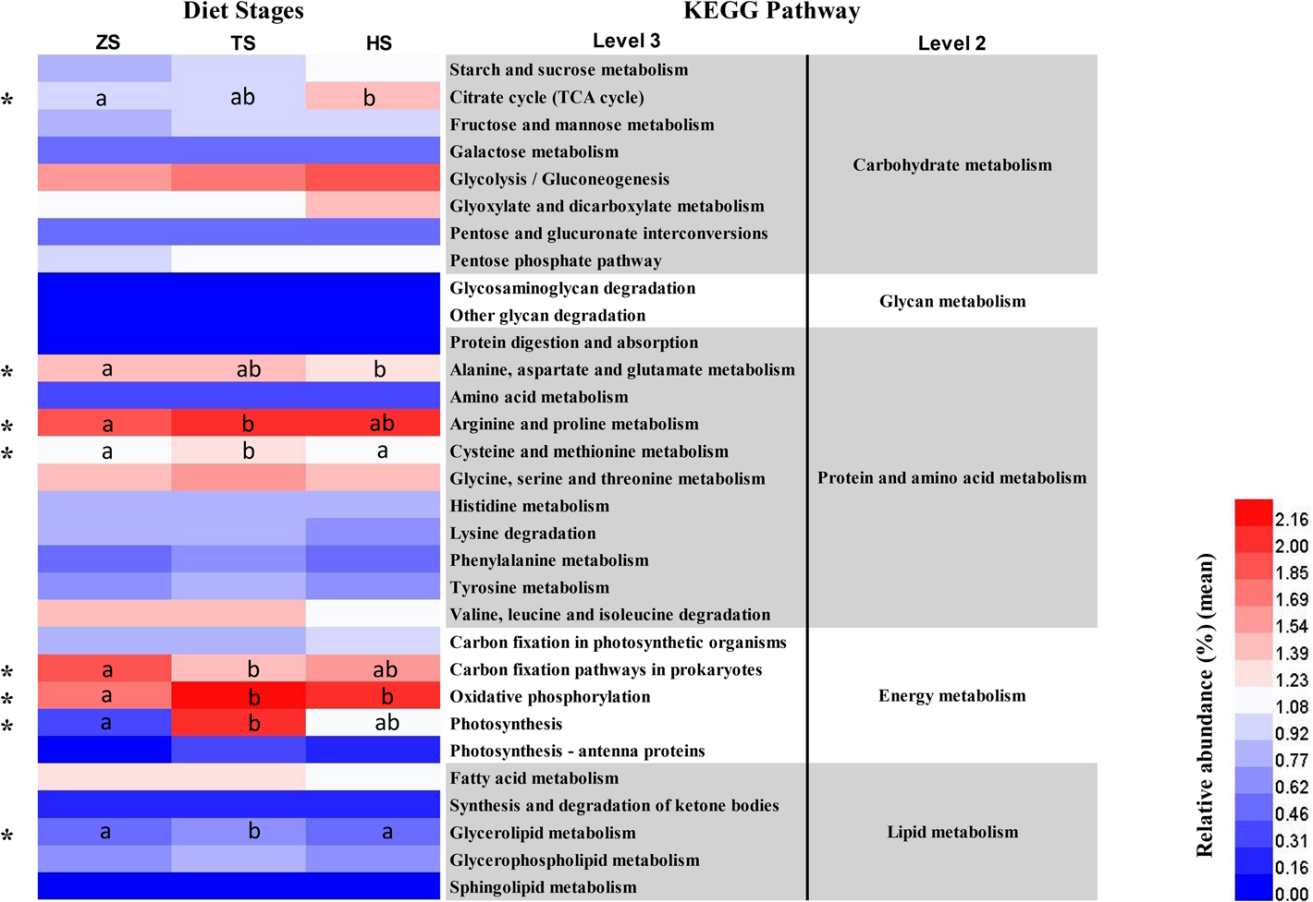
Heat map comparing the abundance of bacterial gene functions among three diet groups. Only KEGG pathways relating to carbohydrate, glycan, protein, amino acid, energy, and lipid metabolism are included in the heat map. Samples sharing the same superscript letters (a, b) indicate no significant difference (*p* > 0.05)

PICRUSt algorithm was performed to predict the fish gut microbiota function based on inferred metagenomes. We identified 223 KEGG pathways, which exhibited similar gene functions but with some difference in abundance among three diet groups; these included pathways relating to carbohydrate, glycan, lipid, protein, energy and amino acid metabolism. As shown in Fig. 7, some of the pathways related to carbohydrate metabolism (starch and sucrose, fructose and mannose, glyoxylate and dicarboxylate, citrate cycle, glycolysis/gluconeogenesis and pentose phosphate) were enriched in HS group, whereas some of the pathways related to protein and amino acid metabolism (alanine, aspartate, glutamate, arginine, proline, glycine, serine and threonine) were enriched in TS group. Additionally, eight pathways were identified, which showed statistically significant differences (*p* < 0.05) in relative abundance among three diet groups.

By comparing the identified genera of this study with the bacterial species known to utilize cellulose [9, 55], we obtained some potential cellulose-degrading bacteria. As shown in Table 2, the proportions of potential cellulolytic bacteria were relatively high in the gut microbiota at transition stages (ranged from 0.85 to 1.72%) and herbivorous stages (ranged from 1.16 to 3.91%), while a relatively low proportion of cellulolytic bacteria was observed at zooplankton-based diet stages (< 1%).

**Table 2:** Relative abundance (%) of potential cellulose-degrading bacteria of the gut microbiota

Bacteria	ZS			TS			HS		
	ZS1	ZS2	ZS3	TS1	TS2	TS3	HS1	HS2	HS3
*Cellulomonas*	0.00	0.05	0.47	0.07	0.00	0.03	0.03	0.58	1.65
*Clostridium*	0.01	0.01	0.05	0.01	0.00	0.78	0.30	1.50	0.89
*Bacillus*	0.11	0.01	0.02	0.01	0.15	0.01	0.02	0.00	0.01
*Ruminococcus*	0.00	0.00	0.00	0.01	1.29	0.00	0.00	0.00	0.03
*Leadbetterella*	0.00	0.00	0.01	0.00	0.00	0.00	0.07	0.09	0.03
*Cytophaga*	0.00	0.01	0.02	0.00	0.00	0.00	0.11	0.16	0.05
*Flavobacterium*	0.01	0.01	0.03	0.06	0.00	0.00	0.16	0.11	0.02
*Achromobacter*	0.00	0.01	0.13	0.06	0.00	0.01	0.01	0.02	0.05
*Vibrio*	0.02	0.01	0.01	0.00	0.00	0.00	0.04	0.00	0.00
*Pseudomonas*	0.01	0.06	0.03	0.40	0.09	0.39	0.14	0.26	0.49
*Brevundimonas*	0.00	0.01	0.03	0.02	0.00	0.44	0.04	0.27	0.42
*Pseudoxanthomonas*	0.00	0.00	0.02	0.20	0.02	0.00	0.04	0.11	0.02
*Methylobacterium*	0.01	0.80	0.14	0.02	0.00	0.05	0.20	0.41	0.26
Total	0.18	0.96	0.96	0.85	1.56	1.72	1.16	3.50	3.91

### Relationship between intestinal microbiota and enzyme activity of gut content

Additional file 8 showed the enzymes activities of gut content in different feeding habit stages. The activities of amylase and cellulase were both significantly higher in HS group than in the other two groups (*p* < 0.05). The activities of lipase and chitinase in TS group were both significantly higher than in ZS and HS groups. Additionally, Fig. 6 reveals the relationship between intestinal microbiota and enzyme activity of gut content. The gut microbiota compositions of fish at early stages of zooplankton-based diet (ZS1 and ZS2) were more related to lipase and chitinase activities but estranged from cellulase and amylase activities. By contrast, the gut microbiota compositions of fish at transition stages (TS1, TS2 and TS3) and herbivorous stages (HS1 and HS2) were more related to cellulase and amylase activities.

## Discussion

Previous studies have mainly focused on the differences in the composition of gut microbiota between different fish species, habitat and trophic categories; recently, researchers have shown an increasing interest in the shift of gut microbiota during host development. However, few studies have reported the impact of dietary transition on the intestinal microbiota of teleost [38, 56]. This study presents a comprehensive survey of the gut microbiota in fish throughout the development of feeding habit, and it provides insight into the relationship among diet transition, digestive enzymes of gut content and intestinal microbiota for the first time.

It is widely acknowledged that the digestive tract of fish is an undifferentiated straight tube with dead-end during the very initial developmental phase, and the nutrition comes from a yolk sac only. The gastrointestinal system develops with the consumption of yolk sac and the initiation of exogenous feeding. Prior to initiation of first feeding, the intestine is open to the surrounding environment, allowing exposure to microbial colonists [7, 17, 57]. In the present study, we observed simple bacterial community structures (diversity and richness) of gut microbiota in blunt snout bream at early stages (ZS1 and ZS2 stages). Thereafter, the diversity and richness of gut microbiota apparently increased from stage ZS3, peaked at stage TS3 and decreased from then on. This result is similar to the previous researches on zebrafish, Atlantic salmon, coho salmon and rainbow trout, which indicated increasing bacterial diversity during the early life stages [7, 17, 58].

The hierarchical cluster analysis and canonical redundancy analysis (RDA) revealed that gut bacterial communities from the fish at different feeding habit stages formed different clusters. This result is consistently related to the clustering result of intestinal content compositions (Additional file 2). These results suggest that the diversity and composition of gut microbiota are greatly affected by the diet composition during the early life stages of blunt snout bream. Previous studies also support the key roles of feeding habit in determining the intestinal microbiota of young and adult fish [17, 59]. Thus, the composition of gut microbiota is closely related to fish feeding habit in all the developmental stages since the fish starts to consume the aliments from environment [2, 11]. Interestingly, we observed a high similarity in the compositions of gut microbiota at stages ZS3 and TS1 (Fig. 4a), although the plant food was not detected until stage TS1 in the present study (Additional file 1). This finding suggests that the adaptability of gut microbiota to the plant-based diet was present before the introduction of plant food. In line with this, a previous study reported that the earliest gut microbiota of human infant can facilitate plant polysaccharide utilization before the introduction of solid food, priming the infant gut for an adult diet [60].

Identification of a core gut microbiome, usually as bacterial taxa shared between 95% of samples tested [61], is crucial for defining a stable and healthy status of the animal gut [2, 62]. Despite the obvious plasticity in the bacterial diversity and composition during different feeding habit stages, the gut microbiota in all the three diet groups were dominated by the two phyla *Proteobacteria* and *Firmicutes*. This result is in line with the findings reported in wild grown blunt snout bream [8, 59], suggesting that those two phyla constitute the ‘core’ microbiota in the gut of blunt snout bream at several life stages regardless of ontogeny and feeding habit transition. Additionally, Fig. 2a revealed that the herbivorous diet favored the presence of *Actinobacteria*. In line with this, the previous research also indicated that *Actinobacteria* was enriched in the gut microbiota of adult *Ctenopharyngodon idellus* (herbivorous) but presented at low abundance in *Aristichthys nobilis* (zooplankton based filter-feeding) [8]. However, *Fusobacteria* was enriched in the gut microbiota of ZS group but remained low in TS and HS groups in the present study. This result differed from earlier studies identifying *Fusobacteria* as part of the core gut microbiota of adult blunt snout bream [8, 59].

A great distinction of microbiota at genus level between diet stages was observed. In this study, *Exiguobacterium* were the most abundant intestinal bacteria of fish at all diet stages. Similarly, great proportions of *Exiguobacterium* were found in the gastrointestinal tract in early life stages of coho salmon fries. In addition, *Cetobacterium* and *Rhizobium* were identified as microbiological markers of gut microbiota at zooplankton-based diet stages and diet transition stages, respectively. Interestingly, the low diversities of bacterial community at ZS1 and ZS2 stages may be due to the high abundances of *Cetobacterium* in the communities, which constituted 44.99% and 43.82% of all sequence reads, respectively. However, this genus was not consistently as abundant in the later stages. In previous studies, *Cetobacterium*, which is capable of inhibiting the growth of other bacterial strains [63], has been found in a variety of freshwater fish species with different trophic categories [56, 64–66]. It is thus likely that *Cetobacterium* is strongly associated with bacterial colonization during early life stages in our study. *Gemmobacter* and *Rhizobium* were significantly enriched in the gut microbiota of fish at transition diet stages. Previous studies reported that *Gemmobacter*, which may be related to lipid degradation, was identified in Pacific white shrimp with high lipid diet [67, 68]. *Rhizobium* was identified in the gastrointestinal tract of *Panaque nigrolineatus* (a wood-eating fish), and this nitrogen-fixing bacterial group is likely to be related to cellulose degradation [69, 70]. Besides, *Acinetobacter* was relatively abundant in ZS and TS groups. Earlier studies indicated that some strains of this genus are potential chitinase-producing or lipase-producing bacteria [25, 71, 72]. Table 2 showed thirteen potential cellulose-degrading bacteria detected in our study. However, no endogenous genes coding cellulose-digesting enzymes were found in the genome of blunt snout bream [73]. Thus, despite the low relative abundance of these bacteria, they might play significant roles in cellulose degradation.

Previous investigations have proposed that intestinal symbiotic microorganisms contribute to fish nutrition [11, 25]. In the present study, we analyzed four main enzyme activities of gut content at different diet stages to explore the possible contribution of gut microbiota to food digestion. As shown in Additional file 8, the activities of amylase and cellulase were both significantly higher in HS group than in the other two groups (*p* < 0.05), while the activities of lipase and chitinase in TS group were both significantly higher than in ZS and HS groups (*p* < 0.05). It suggests that the enzyme activity of gut content was affected by dietary manipulation. However, the activities of these four enzymes were found to be relatively low in the gut content of ZS group. In line with this, previous studies also indicated that the activities of exogenous and endogenous enzymes in fish were relatively low during the early life stages [74, 75]. In this study, although it is unclear whether these gut content enzymes are produced by the host or by the gut microbiota, the canonical redundancy analysis (RDA) clearly revealed that the feeding habits strongly affected the intestinal microbiota and the digestive enzyme activities of gut content. Additionally, PICRUSt predictions of metagenome function demonstrate that some of the pathways related to carbohydrate metabolism were enriched in HS group, while some of the pathways related to protein and amino acid metabolism were enriched in TS group. This result suggests that the metabolic capacity of gut microbiota was affected by feeding habit.

In our study, the rearing strategy makes the fish grow in a nearly natural state, so the variations of the gut microbiota during the dietary transition are truly reflected. Additionally, we used pooled samples to eliminate the inter-individual differences in the intestinal bacterial community. The sequencing data from each sub-stage was treated as biological replicates in the three diet groups when we conducted significance tests on the gut microbiota among three coarse stages of feeding habit. This approach is considered to reflect the state of microbiota more exactly because every three sampling time points spanned the course of each feeding habit stage. Unfortunately, due to a very small amount of intestinal contents obtained, no technical replicate was used in the significance tests on gut microbiota. Further, a limitation of this study is the inability to distinguish resident bacteria and transient bacteria derived from food in the gut of blunt snout bream. Therefore, what roles the resident bacteria perform in the gut microbiota and how these bacteria interact with their hosts need to be clarified in the future.

## Conclusions

This study is the first comprehensive and valuable analyses of gut microbiota and digestive enzymes of gut content in fish during periods of feeding habit transition, which enabled us to better understand the relationship among gut microbiota, nutrition metabolism and feeding habits in vertebrate. Further, our study provides a reference for future studies investigating the metabolic adaption of herbivorous fish to shift to a vegetarian diet during their life history.

## Additional files

Additional file 1: Mean proportion ± SE of each prey item in intestinal contents derived from nine diet sub-stages. Samples sharing the same superscript letters (^a, b, c, d, e^) indicate no significant difference (*p* > 0.05) by Nemenyi test. (DOCX 518 kb)

Additional file 2: The cluster dendrogram of intestinal contents derived from nine diet sub-stages. Cluster dendrogram used Ward’s method for compositions of intestinal contents from blunt snout bream at nine diet sub-stages. (DOCX 92 kb)

Additional file 3: Rarefaction curves estimating the richness (at a 97% similarity level) of intestinal microbiota derived from nine diet sub-stages. The vertical axis shows the number of OTUs that would be detected after sampling the sequences, of which the number was shown on the horizontal axis. (DOCX 79 kb)

Additional file 4: Species accumulation curves determining whether the number of sequenced samples is sufficient. The vertical axis shows the number of OTUs that would be detected after sampling the samples, of which the number was shown on the horizontal axis. (DOCX 93 kb)

Additional file 5: Rank-abundance curves of OTUs derived from nine diet sub-stages. Rank abundance distribution curves showing the OTUs within each category ranked according to their abundance in the corresponding combined OTU sequence data set. (DOCX 148 kb)

Additional file 6: Rank-abundance curves of OTUs derived from the three diet groups. Rank abundance distribution curves showing the OTUs within each category ranked according to their abundance in the corresponding combined OTU sequence data set. (DOCX 124 kb)

Additional file 7: Comparisons in the relative abundance of bacteria at phylum, order, family and genus levels among three diet groups (ZS, TS and HS). The diet groups sharing the same superscript letters (^a, b, c^) indicate no significant difference (*p* > 0.05). (DOCX 495 kb)

Additional file 8: The means (mean ± SE) of enzymes activities of gut content at different feeding habit stages. Diet groups sharing the same letters (a, b) indicate no significant difference (*p* > 0.05). ANOVA was followed by Tukey’s test. (DOCX 28 kb)

## Abbreviations

d.p.f.: Day post fertilization
DGGE: Denaturing gradient gel electrophoresis;
HS: The herbivorous diet stage
KEGG: Kyoto Encyclopedia of Gene and Genomes
LEfSe: Linear discriminant analysis effect size
NCBI: National Center for Biotechnology Information
NGS: Next-generation sequencing
OTUs: Operational Taxonomic Units
PICRUSt: Phylogenetic Investigation of Communities by Reconstruction of Unobserved States
RDA: Redundancy analysis
SRA: Short Read Archive
T-RFLP: Terminal restriction fragment length polymorphism
TS: The diet transition stage
ZS: The zooplankton-based diet stage
