# Supplementary figures and images for: The variation profile of intestinal microbiota in blunt snout bream (Megalobrama amblycephala) during feeding habit transition
Source: BMC Microbiol. 2018 Sep 3;18:99. doi: 10.1186/s12866-018-1246-0 (PMC6122550; doi:10.1186/s12866-018-1246-0)

**Additional file 1**


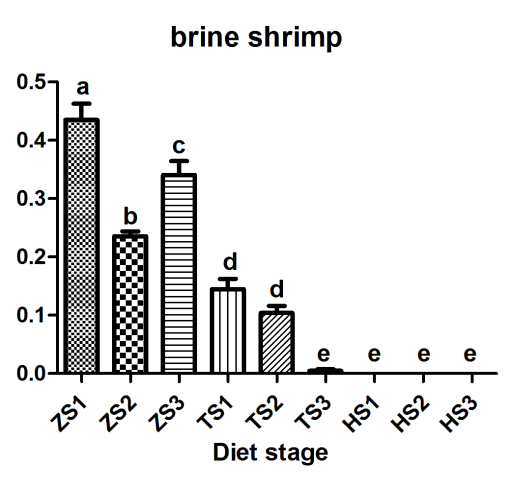

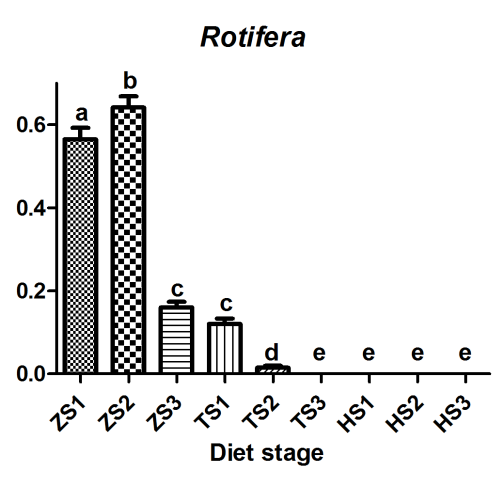


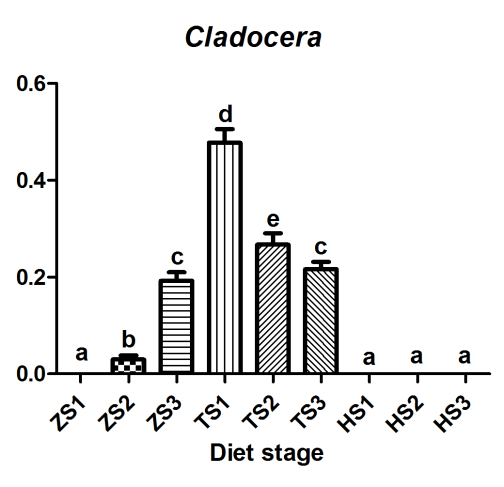

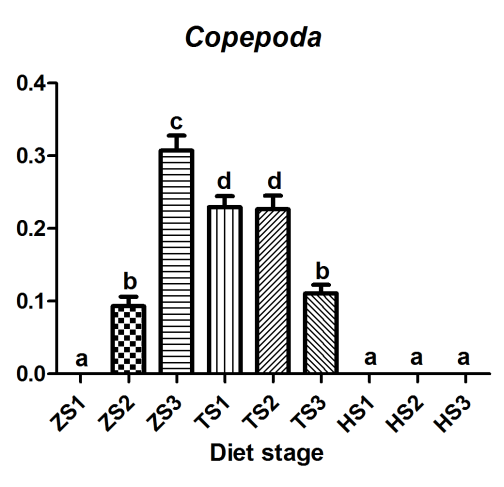


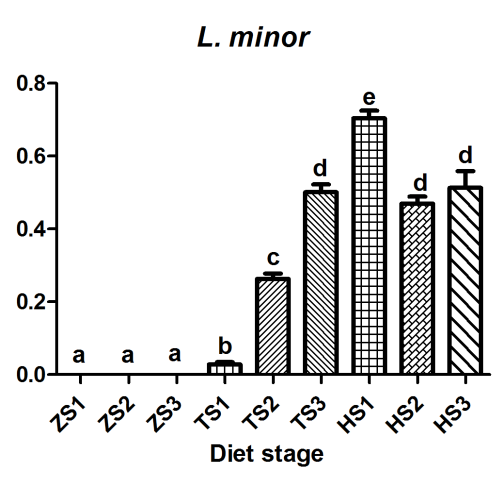

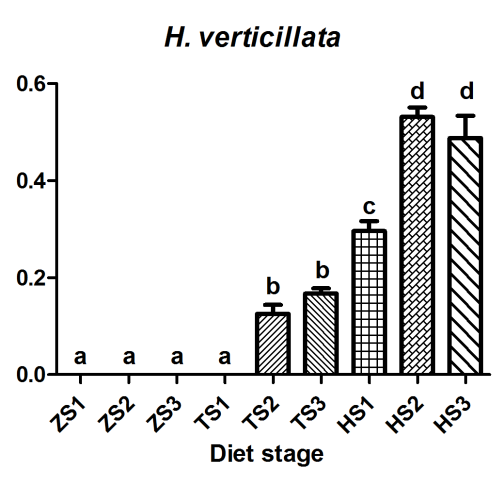

Supplement: Supplementary file 1 — Mean proportion ± SE of each prey item in intestinal contents derived from nine diet sub-stages. Samples sharing the same superscript letters (a, b, c, d, e) indicate no significant difference (p > 0.05) by Nemenyi test. (DOCX 518 kb) [file 12866_2018_1246_MOESM1_ESM.docx]

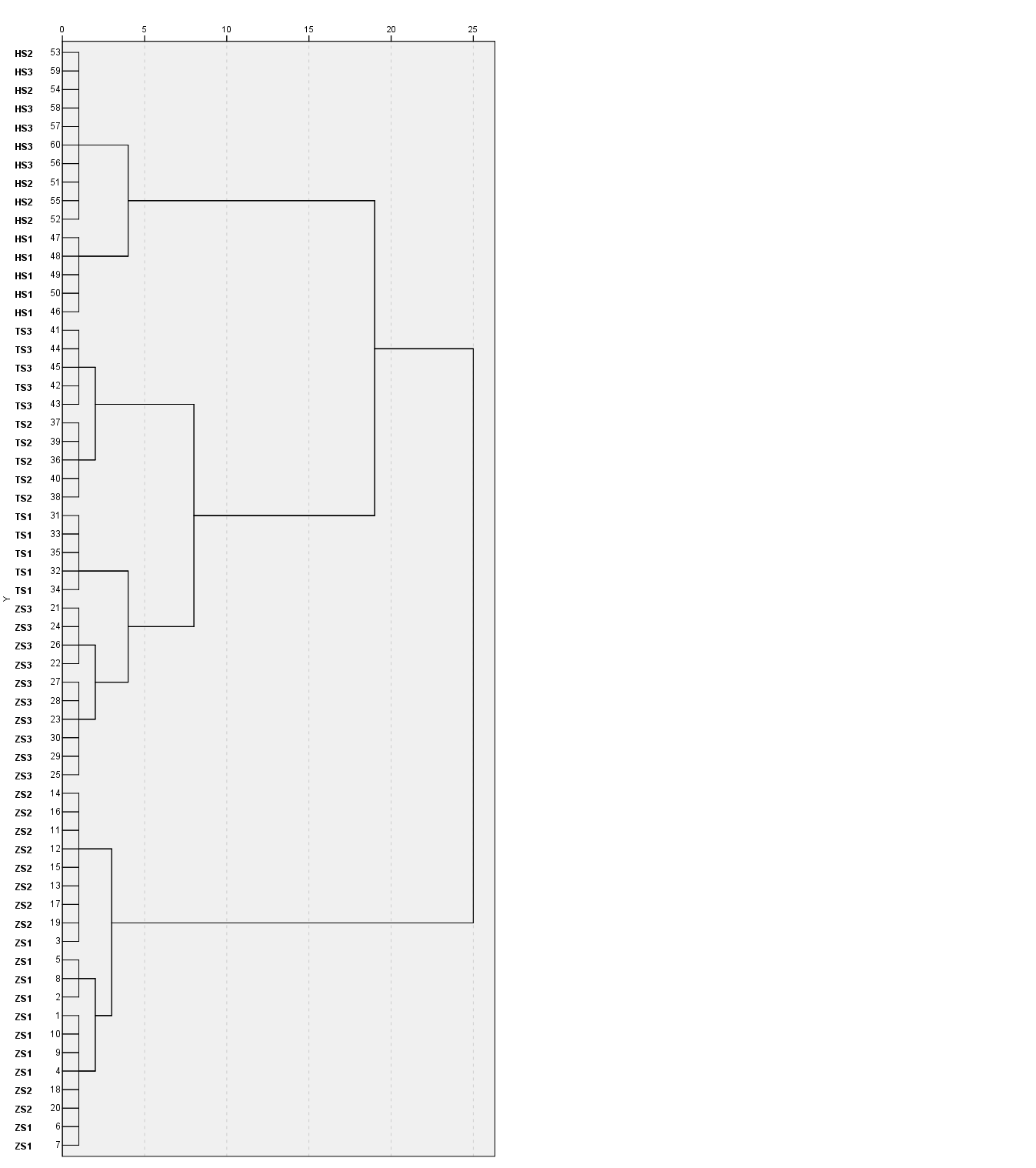
**Additional file 2**

**Rescaled Distance Cluster Combine**

Supplement: Supplementary file 2 — The cluster dendrogram of intestinal contents derived from nine diet sub-stages. Cluster dendrogram used Ward’s method for compositions of intestinal contents from blunt snout bream at nine diet sub-stages. (DOCX 92 kb) [file 12866_2018_1246_MOESM2_ESM.docx]

**
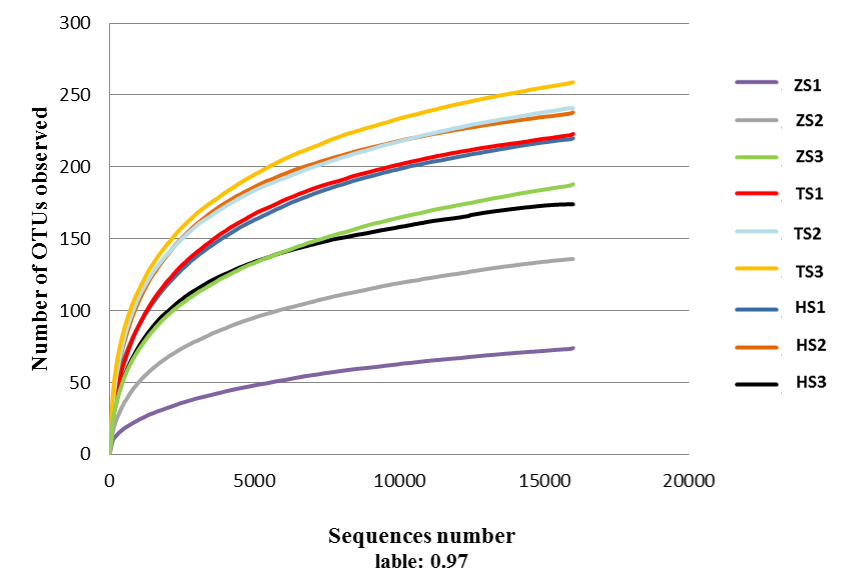
Additional file 3**

Supplement: Supplementary file 3 — Rarefaction curves estimating the richness (at a 97% similarity level) of intestinal microbiota derived from nine diet sub-stages. The vertical axis shows the number of OTUs that would be detected after sampling the sequences, of which the number was shown on the horizontal axis. (DOCX 79 kb) [file 12866_2018_1246_MOESM3_ESM.docx]

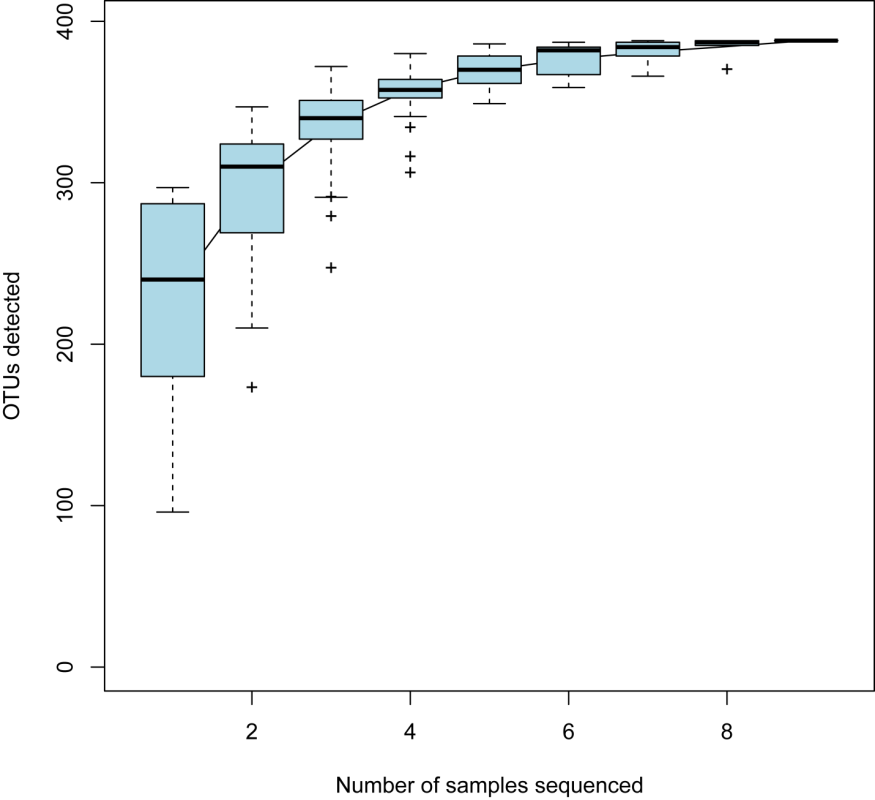
**Additional file 4**

Supplement: Supplementary file 4 — Species accumulation curves determining whether the number of sequenced samples is sufficient. The vertical axis shows the number of OTUs that would be detected after sampling the samples, of which the number was shown on the horizontal axis. (DOCX 93 kb) [file 12866_2018_1246_MOESM4_ESM.docx]

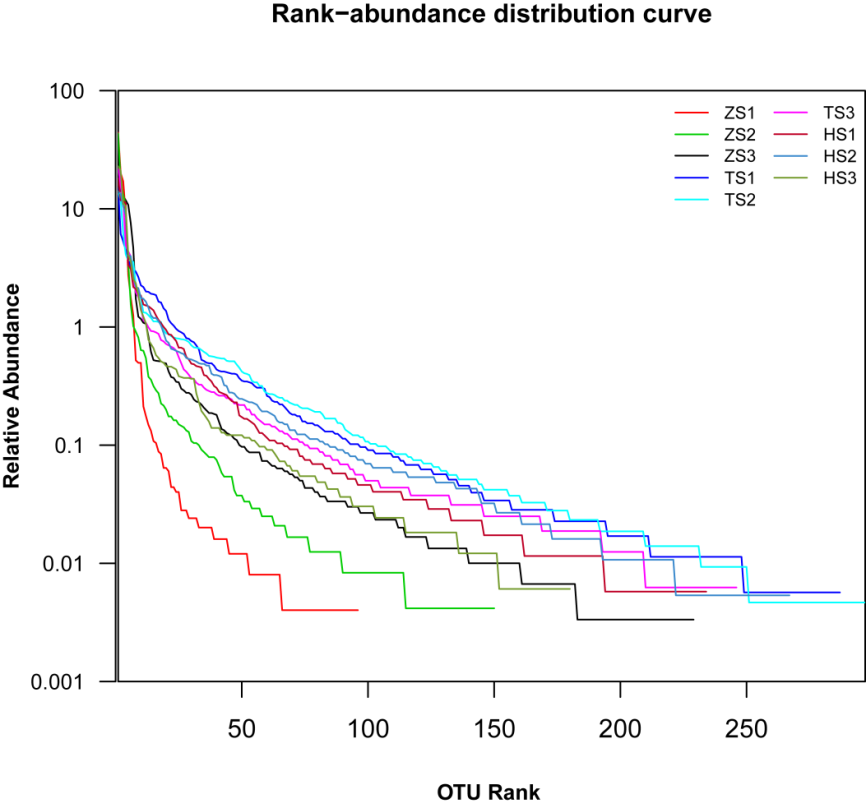
**Additional file 5**

Supplement: Supplementary file 5 — Rank-abundance curves of OTUs derived from nine diet sub-stages. Rank abundance distribution curves showing the OTUs within each category ranked according to their abundance in the corresponding combined OTU sequence data set. (DOCX 148 kb) [file 12866_2018_1246_MOESM5_ESM.docx]

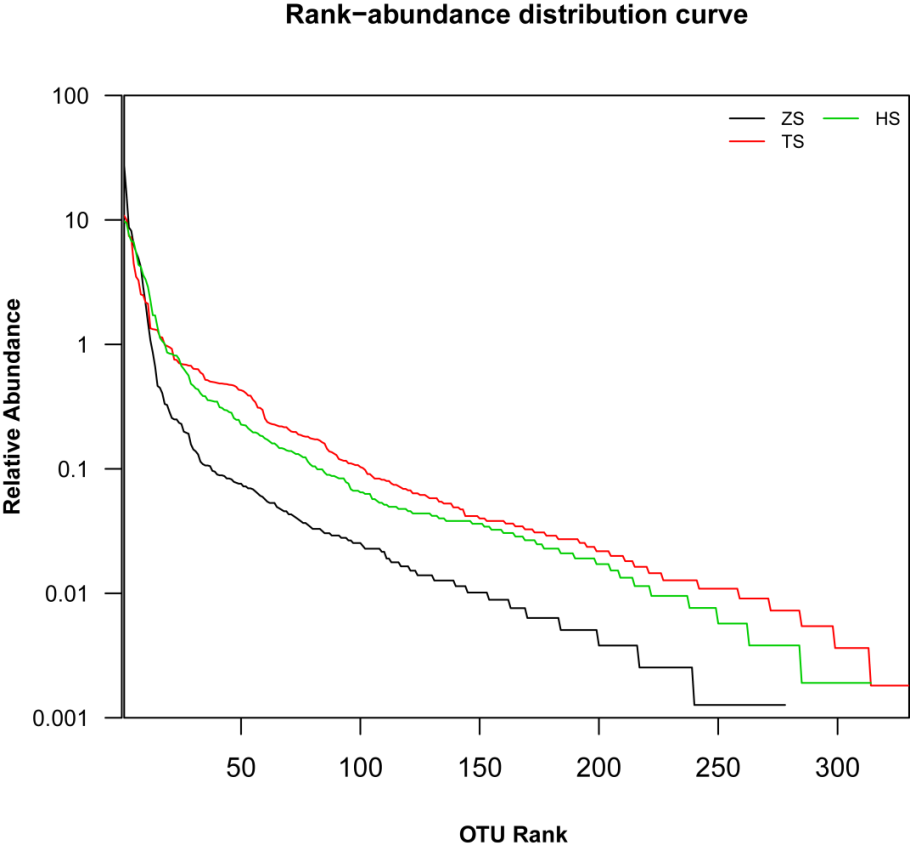
**Additional file 6**

Supplement: Supplementary file 6 — Rank-abundance curves of OTUs derived from the three diet groups. Rank abundance distribution curves showing the OTUs within each category ranked according to their abundance in the corresponding combined OTU sequence data set. (DOCX 124 kb) [file 12866_2018_1246_MOESM6_ESM.docx]

**
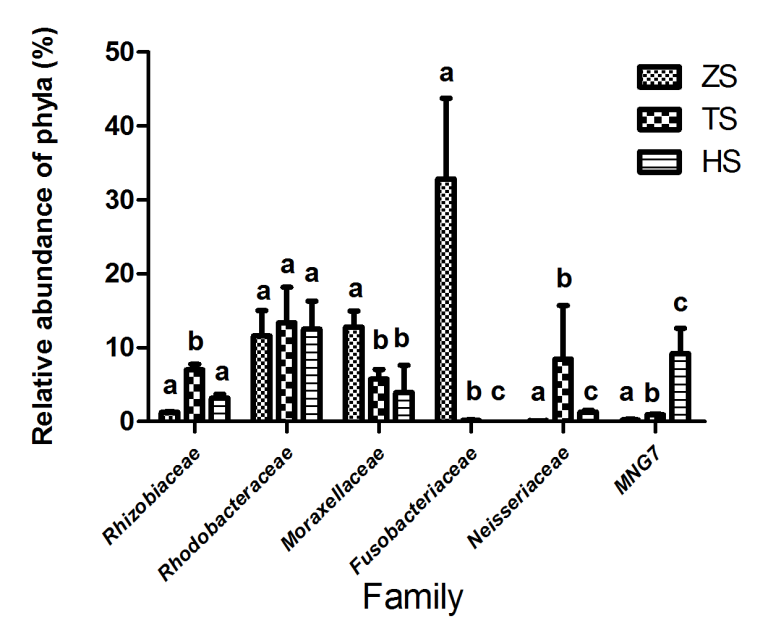

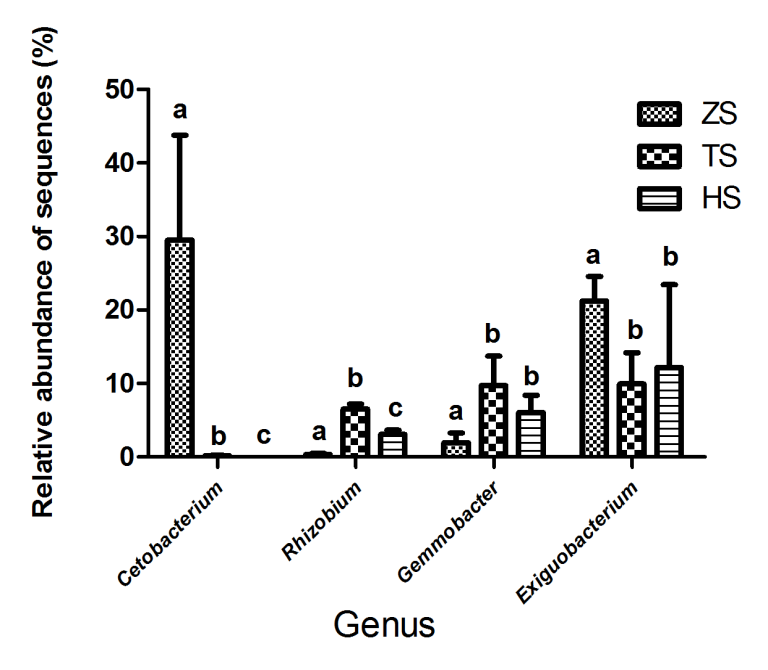

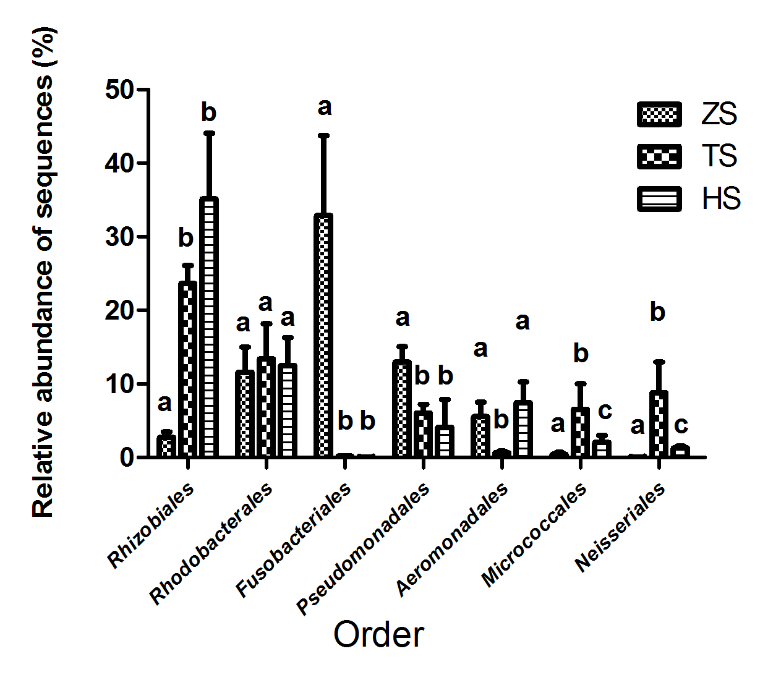

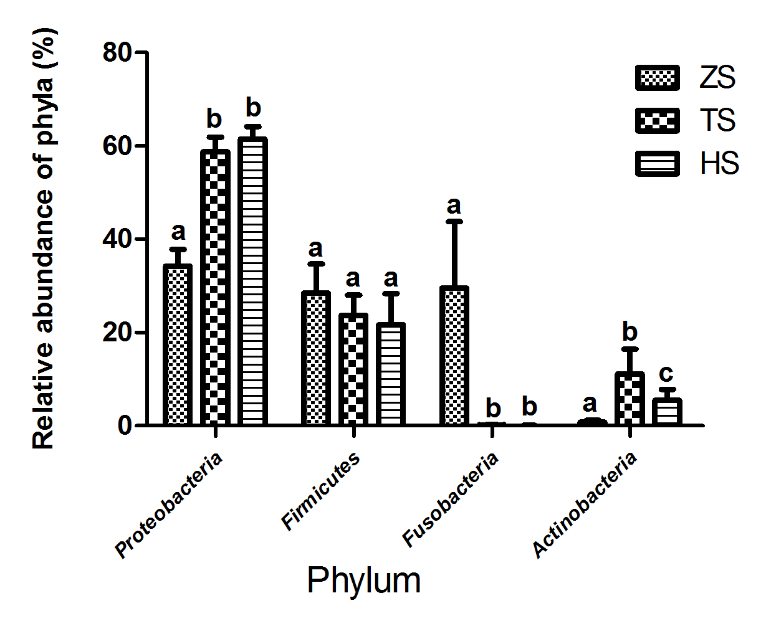
Additional file 7**

Supplement: Supplementary file 7 — Comparisons in the relative abundance of bacteria at phylum, order, family and genus levels among three diet groups (ZS, TS and HS). The diet groups sharing the same superscript letters (a, b, c) indicate no significant difference (p > 0.05). (DOCX 495 kb) [file 12866_2018_1246_MOESM7_ESM.docx]
