# Supplementary material for: The variation profile of intestinal microbiota in blunt snout bream (Megalobrama amblycephala) during feeding habit transition
Source: BMC Microbiol. 2018 Sep 3;18:99. doi: 10.1186/s12866-018-1246-0 (PMC6122550; doi:10.1186/s12866-018-1246-0)
Supplement: Supplementary file 8 — The means (mean ± SE) of enzymes activities of gut content at different feeding habit stages. Diet groups sharing the same letters (a, b) indicate no significant difference (p > 0.05). ANOVA was followed by Tukey’s test. (DOCX 28 kb) [file 12866_2018_1246_MOESM8_ESM.docx]

**Additional file 8**
